# Metformin blocks MYC protein synthesis in colorectal cancer via mTOR‐4EBP‐eIF4E and MNK1‐eIF4G‐eIF4E signaling

**DOI:** 10.1002/1878-0261.12384

**Published:** 2018-10-15

**Authors:** Peng Shen, Lucas C. Reineke, Erik Knutsen, Meng Chen, Martin Pichler, Hui Ling, George A. Calin

**Affiliations:** ^1^ Department of Oncology Nanfang Hospital Southern Medical University Guangzhou China; ^2^ The First School of Clinical Medicine Southern Medical University Guangzhou China; ^3^ Department of Experimental Therapeutics The University of Texas MD Anderson Cancer Center Houston TX USA; ^4^ Department of Molecular Physiology and Biophysics Baylor College of Medicine Houston TX USA; ^5^ Department of Medical Biology Faculty of Health Sciences UiT – The Arctic University of Norway Tromsø Norway; ^6^ Division of Oncology Research Unit of Non‐Coding RNA and Genome Editing in Cancer Medical University of Graz Austria; ^7^ The Center for RNA Interference and Non‐Coding RNAs The University of Texas MD Anderson Cancer Center Houston TX USA; ^8^ Present address: Cell & Gene Therapy, Bioverativ A Sanofi Company Waltham MA 02451 USA

**Keywords:** cell cycle, colorectal cancer, metformin, mTOR, MYC, protein synthesis

## Abstract

The antidiabetic drug metformin has been associated with reduced colorectal cancer (CRC) risk and improved prognosis of CRC patients. However, the detailed mechanisms underlying such beneficial effects remain unknown. In this study, we aimed to evaluate metformin activity in CRC models and unveil the underlying molecular mechanisms. We showed that metformin inhibits CRC cell proliferation by arresting cells in the G1 phase of the cell cycle and dramatically reduces colony formation of CRC cells. We discovered that metformin causes a robust reduction of MYC protein level. Through the use of luciferase assay and coincubation with either protein synthesis or proteasome inhibitors, we demonstrated that regulation of MYC by metformin is independent of the proteasome and 3′ UTR‐mediated regulation, but depends on protein synthesis. Data from polysome profiling and ribopuromycylation assays showed that metformin induced widespread inhibition of protein synthesis. Repression of protein synthesis by metformin preferentially affects cell cycle‐associated proteins, by altering signaling through the mTOR‐4EBP‐eIF4E and MNK1‐eIF4G‐eIF4E axes. The inhibition of MYC protein synthesis may underlie metformin's beneficial effects on CRC risk and prognosis.

**Abbreviations**
CRC: colorectal cancer
UTR: untranslated region

## 1 Introduction

Colorectal cancer (CRC) is the third most frequent cancer accounting for 8% of new cancer cases and the third leading cause of cancer‐related mortalities in the United States (Siegel *et al*., 2017b). Moreover, the CRC incidence and mortality rate in young adults below 50 years of age increased by 22% and 13%, respectively, from 2000 to 2013 in the United States (Siegel *et al*., 2017a). The upward trend of CRC in younger individuals urges discovery of novel and effective prevention strategies.

Metformin is the first‐line treatment for people with type 2 diabetes mellitus. Epidemiological studies suggest metformin treatment decreases cancer risk in diabetic patients (Bodmer *et al*., 2010; Currie *et al*., 2009; Evans *et al*., 2005; Tsai *et al*., 2014). More recently, a randomized phase 3 clinical trial showed that metformin is equally effective in preventing recurrence of colorectal adenoma and polyps in patients without diabetes (Higurashi *et al*., 2016). The beneficial effect of metformin in CRC is supported by experimental evidence including reduction of spontaneous intestinal polyp growth in Apc^Min/+^ mice and synergistic activity with chemotherapeutic drugs in controlling CRC growth (Tomimoto *et al*., 2008). Currently, multiple clinical trials are evaluating the effect of combinations of metformin with standard chemotherapeutics in CRC treatment. However, the molecular mechanism contributing to metformin's beneficial effects in CRC remains elusive (Thent *et al*., 2017).

In this report, we evaluated the effects of metformin on CRC growth and characterized the underlying molecular mechanisms therein. We showed that metformin reduces CRC cell proliferation and colony formation by arresting cells in the G1 phase of the cell cycle. We identified the MYC oncogene as a molecular target of metformin and demonstrated robust inhibition of MYC protein synthesis by metformin without affecting MYC mRNA levels. We further demonstrated a broad effect of metformin on protein synthesis, with a preference for cell cycle‐related proteins, and alteration of known pathways affecting protein synthesis: AMPK‐mTOR‐4EBP‐eIF4E and MNK1‐eIF4G‐eIF4E.

## 2 Materials and methods

### 2.1 Cell lines and culture

The microsatellite instable (MSI) HCT116 and DLD1, the microsatellite stable (MSS) HT29 and COLO320, and HEK293 cells were obtained from the American Type Culture Collection and validated by the Characterized Cell Line Core at The University of Texas MD Anderson Cancer Center using STR DNA fingerprinting. The p53‐deficient HCT116 cells (named HCT116 p53^−/−^) were a gift from Bert Vogelstein's laboratory (Bunz *et al*., 1999). Cells were maintained in Dulbecco's modified Eagle's medium supplemented with 10% fetal bovine serum. All cultures were grown in 5% CO_2_ at 37 °C.

### 2.2 Reagents

The list of reagents is provided in Table S1. Briefly, metformin hydrochloride was purchased from Abcam (Cambridge, MA, USA); cycloheximide, AICAR, and dorsomorphin were purchased from Sigma‐Aldrich (St. Louis, MO, USA); MG‐132, MHY1485, and rapamycin were purchased from EMD Millipore (Billerica, MA, USA).

### 2.3 Cell viability assay

Equal numbers of cells were seeded in 96‐well plates and allowed to adhere overnight. Cells were then incubated in the presence of solvent control or the indicated concentrations of metformin. Cell number was assessed using the Cell Counting Kit 8 (Dojindo Molecular Technologies, Gaithersburg, MD, USA), and the absorbance was measured at 450 nm by spectrophotometry following the manufacturer's instructions.

### 2.4 Cell cycle analysis

Equal numbers of cells were seeded in 10‐cm dish to reach 60% confluence after overnight incubation. Cells were treated with metformin or solvent control for 48 h and fixed in 70% ethanol overnight at 4 °C. The fixed cells were stained with 50 μg·mL^−1^ propidium iodide (PI), containing 20 μg·mL^−1^ of RNase A for 15 min, and subsequently subjected to flow cytometric analysis.

### 2.5 Colony‐forming assay

Cells were trypsinized and seeded in 6‐well plate at 500 or 1000 cells/well. After overnight incubation, cells were exposed to metformin or vehicle control for 10 days. The cells were fixed with absolute methanol and stained with 1% crystal violet (Sigma‐Aldrich) (Crowley *et al*., 2016).

### 2.6 RNA isolation, cDNA synthesis, and real‐time quantitative PCR

RNA isolation, cDNA synthesis, and PCR were performed as previously described (Ohtsuka *et al*., 2016). Primer sequences are available in Table S2.

### 2.7 Protein extraction and immunoblotting

Cells were lysed in Laemmli sample buffer (Bio‐Rad Laboratories, Hercules, CA, USA) containing protease inhibitor cocktail (Sigma) and phosphatase inhibitor cocktail 2 (Sigma). The immunoblotting analysis was performed as previously described (Ohtsuka *et al*., 2016). β‐Actin was used to ensure equivalent protein loading. The immunoblotting images were semiquantified using the imagej software (https://imagej.nih.gov/ij/), normalized to the signal of β‐actin, and further normalized with the control set as 1. See Table S1 for the list of antibodies.

### 2.8 Protein synthesis assay

1x10^6^ cells were seeded and left to attach overnight before specific treatments for 24 h in glucose‐free media. Samples were further processed according to the manufacturer's instructions using Protein Synthesis Assay Kit (601100, Cayman Chemical, Ann Arbor, MI, USA). Cells were then resuspended with OPP Working Solution for 30 min at 37 °C, washed and stained with the 5 FAM‐Azide Staining Solution, and analyzed by flow cytometry (FC500, Beckman Coulter, Brea, CA, USA).

### 2.9 Polysome profiling assay

Briefly, metformin and control‐treated cells were grown to ~70% confluence. Cells were treated with 100 μg·mL^−1^ cycloheximide at 37 °C for 15 min prior to harvesting. Cells were lysed in lysis buffer (Pereboom *et al*., 2014), using homogenization on ice. Lysates were cleared by centrifugation at 1200 ***g*** for 10 min, and equal OD260 units were loaded onto a 17–50% sucrose gradient. Sucrose gradients were centrifuged for 2 h at 178 305 ***g*** in a Beckman SW41 rotor (Beckman Coulter, Indiana, USA) at 4 °C prior to fractionation. Fractionation was performed on an ISCO UV spectrophotometer and gradient fractionator (Teledyne ISCO, Nebraska, USA). Data were collected with labworks software (Lehi, UT, USA). Postcollection data analysis was performed in Microsoft Excel and graphpad prism 7 (La Jolla, CA, USA).

### 2.10 3′‐UTR luciferase assay

The miRNA 3′ UTR target clones including MYC (NM_002467.4) 3′‐UTR dual‐luciferase reporter (HmiT067350‐MT05) and control dual‐luciferase reporter (CmiT000001‐MT05) were purchased from GeneCopoeia (Rockville, MD, USA). Cells were transfected with the reporter plasmid using Lipofectamine^®^ 2000 (Thermo Fisher Scientific, Waltham, MA, USA) for 24 h and then incubated with fresh standard cell culture medium containing vehicle or metformin for another 24 h. The cell culture medium was collected and analyzed using Secrete‐Pair™ Dual Luminescence Assay Kit (GeneCopoeia). The secreted *Gaussia* Luciferase activity was normalized by the activity of the constitutively expressed, secreted alkaline phosphatase from the same plasmid. The normalized *Gaussia* luciferase activity in the MYC 3′‐UTR reporter was further normalized by that in the control plasmid.

### 2.11 Plasmid and virus generation

The lentivirus expression plasmids including pLOC‐MYC (Clone ID: PLOHS_100008545) and pLOC‐RFP (control vector) were purchased from Dharmacon (Lafayette, CO, USA). We produced virus soup in 293 FT cells according to the instructions of the manufacturer and used it to induce MYC expression in CRC cells.

### 2.12 Reverse‐phase protein array

HT29, HCT116, HCT116 P53^−/−^, and DLD1 cells were seeded in 100‐mm dishes at 3 million cells per dish with standard cell culture medium containing 25 mm glucose. The next day, cells were washed with PBS and incubated in fresh glucose‐free cell culture medium in the presence of vehicle control or 2 mm metformin for 24 h. Biological duplicates were used for each treatment in all cell lines. Cells were lysed in 4 × SDS sample buffer (40% glycerol, 8% SDS, 0.25 m Tris/HCl, 10% 2‐mercaptoethanol, pH 6.8). The concentration of proteins was adjusted to 1.0 μg·μL^−1^ before submission to the reverse‐phase protein array (RPPA) core facility at MD Anderson Cancer Center for array and analysis. Briefly, serial dilutions of samples were arrayed on nitrocellulose‐coated slides and run against 302 antibodies. Spot density was determined by Array Pro, and protein concentration was determined by super curve fitting. The relative protein expression after normalization was used for comparison between vehicle control and metformin treatment.

### 2.13 Statistical analysis

The data are presented as mean ± standard deviation. Greater than or equal to 3 independent experiments were conducted, and significance was assessed using Student's *t*‐tests. *P*‐values <0.05 were considered statistically significant. Statistical analyses were performed with GraphPad Prism 7 (La Jolla, CA, USA).

## 3 Results

### 3.1 Metformin inhibits CRC cell growth and colony formation

**Figure 1:**
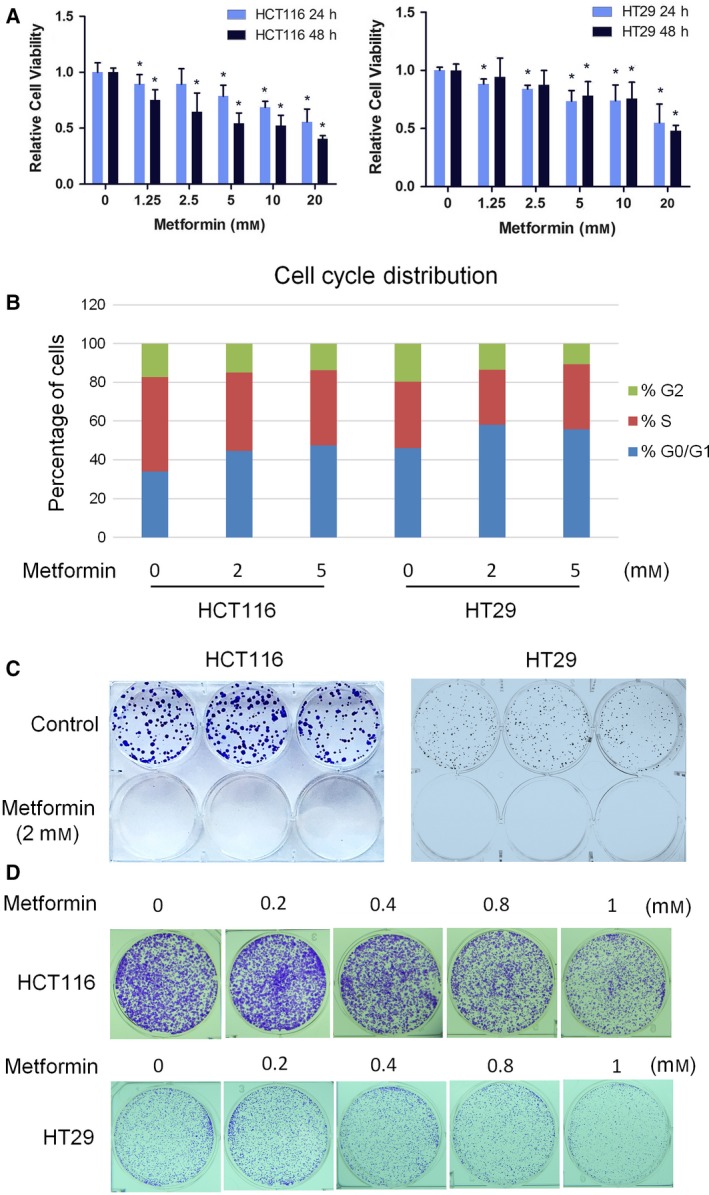
Metformin suppresses CRC cell growth and colony formation. (A) Metformin inhibits HCT116 and HT29 cell growth in a dose‐dependent manner. (B) Metformin arrests the HCT116 and HT29 cells at G1 phase. (C) Metformin at 2 mm completely abrogates the colony formation ability of HCT116 and HT29 cells. (D) Metformin reduces colony formation of CRC cells in a dose‐dependent fashion. The proliferation data are presented as the means ± SD of values obtained in 3 independent experiments. Student's *t*‐test was used to assess significance relative to vehicle control. *P <* *0.05.

We first evaluated the effect of metformin on CRC cellular growth by means of a CCK‐8 assay. Treatment of MSI HCT116 and MSS HT29 cells with metformin results in a dose‐dependent inhibition of cell growth, with significant differences observed at a concentration of 5 mm metformin at both 24 h and 48 h (Fig. 1A). We observed similar cell growth inhibition in HCT116 p53^−/−^ and DLD1 CRC cells (Fig. S1). To address whether the inhibitory effect is due to reduced proliferation or increased cell death, we performed PI staining for initial analysis of apoptosis and cell cycle distribution. Flow cytometric analysis revealed a clear cell cycle arrest in G1, with a concomitant reduction of S‐phase cells (Fig. 1B). No induction of apoptosis in sub‐G1 phase cells was observed after 48‐h metformin treatment (Fig. S2). To further test the cytotoxic effects of metformin on CRC cell growth, we performed a colony formation assay to determine the capacity of a single cell to grow into a colony. At a concentration of 2 mm metformin, which minimally reduced cell growth in the CCK8 proliferation assay, metformin completely abrogated colony formation of HCT116 and HT29 cells (Fig. 1C). The effect of metformin on colony formation was dose dependent (Fig. 1D), and similar effects were observed in additional CRC cell lines (Fig. S3). Together, these data suggest a strong inhibitory effect of metformin on CRC cell growth.

### 3.2 Metformin reduces MYC protein expression

**Figure 2:**
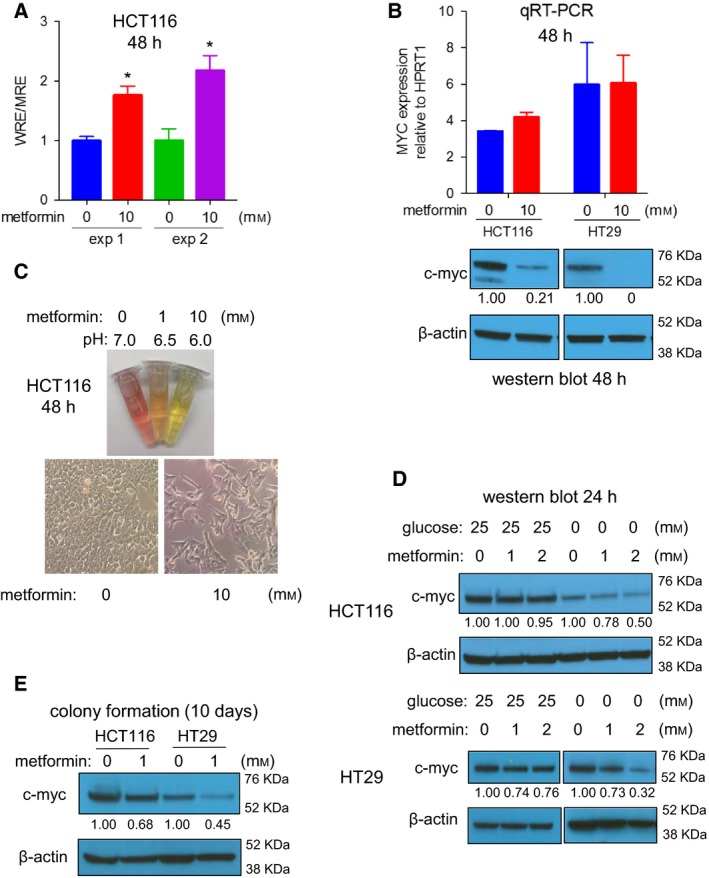
Metformin reduces MYC protein expression in CRC cells. (A) Metformin increases Wnt activity as evaluated by TOPFLASH luciferase reporter. (B) Metformin dramatically reduces MYC protein expression without affecting the MYC RNA level. (C) Metformin promotes glycolysis, as shown by acidic medium, and change of cell morphology into spindle‐like shape. (D) Metformin reduces MYC protein expression in both standard culture condition and glucose‐free condition. (E) Metformin diminishes MYC protein expression in colony formation cells. The luciferase data in (A) are 2 independent experiments (exp1 and exp2) in quadruplicate, and data represent means ± SD. The qRT‐PCR data in (B) are presented as the means ± SD (HCT116, *n* = 2; HT29, *n* = 4). Student's *t*‐test was used to assess significance. *P <* *0.05.

Wnt signaling is an essential factor in CRC initiation and proliferation (Cancer Genome Atlas, 2012; Liu *et al*., 2000; Phelps *et al*., 2009). We evaluated the possibility that metformin inhibits CRC growth by reducing Wnt signaling. To our surprise, we observed an increase in Wnt activity in a TOPFLASH luciferase Wnt reporter assay, instead of the expected decrease, following treatment with 10 mm metformin (Fig. 2A). In concordance with the Wnt activity data, 10 mm metformin did not reduce expression of MYC mRNA, a classical Wnt target gene, in all CRC cell lines tested except for RKO cells (Figs 2B and S4). However, 10 mm metformin caused almost complete depletion of MYC protein in all tested CRC cell lines (Figs 2B and S4). Cell growth inhibition by metformin is accompanied by a spindle‐like morphological shape and acidification of the media (Fig. 2C). Cancer cells are known to enhance glycolysis in the presence of metformin in an attempt to compensate for energy loss following metformin inhibition of the mitochondrial respiration chain complex (Marini *et al*., 2016). To determine whether this compensation causes the reduction of MYC protein, we examined MYC protein during metformin treatment in either standard culture conditions containing 25 mm of glucose, or glucose‐free culture conditions. The inhibitory effect of metformin on MYC protein is also observed under glucose‐free conditions, with a clear MYC reduction under 2 mm metformin concentration (Fig. 2D). These data suggest that MYC reduction by metformin is independent of glucose and is not the consequence of enhanced glycolysis. We next tested whether MYC reduction occurs in colony formation assays. Because 2 mm metformin completely blocked colony formation 10 days after cell seeding and no material could be obtained, we examined MYC expression in colonies formed in the presence of a lower concentration of metformin (1 mm). Consistently, we detected lower MYC protein expression in the colonies exposed to metformin, in contrast to those without metformin exposure (Fig. 2E). Together, these data suggest a robust post‐transcriptional regulation of MYC by metformin.

### 3.3 Metformin blocks MYC protein synthesis

**Figure 3:**
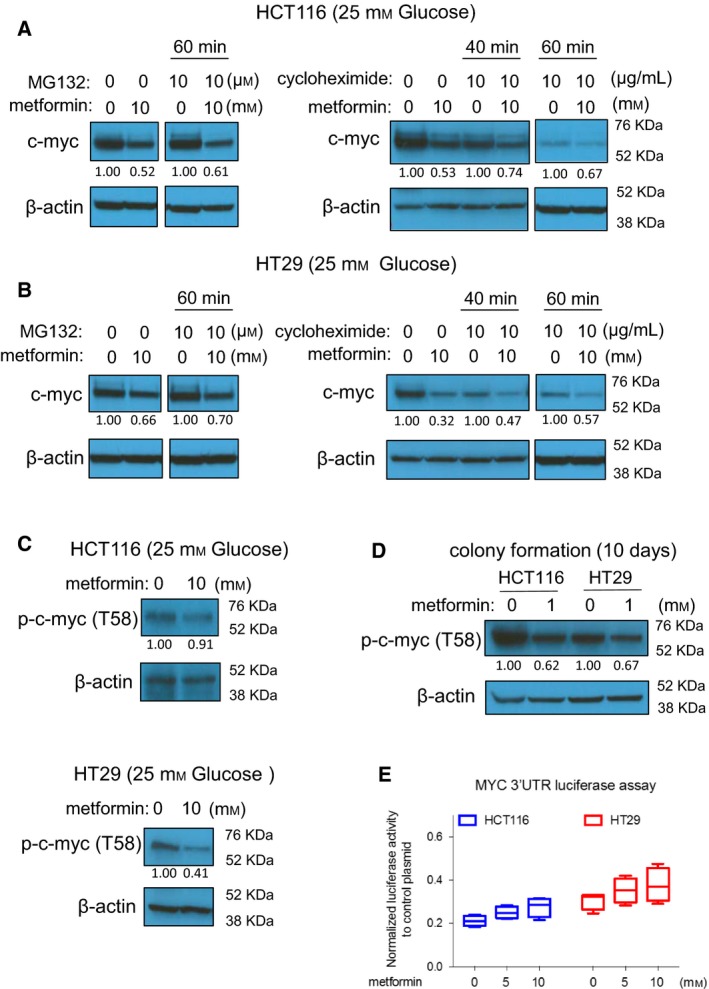
Metformin blocks MYC protein synthesis in standard culture conditions. (A,B) Metformin‐induced MYC reduction is neither rescued by the proteasome inhibitor MG132, nor accelerated by the protein synthesis inhibitor CHX in HCT116 cells (A) and in HT29 cells (B). In A and B, cells were treated with metformin for 24 h (10 mm), in the presence or absence of MG132 (10 μM). (C,D) Metformin does not enhance the MYC phosphorylation at threonine 58 in 24 h treatment (C) or in colony formation samples (D). (E) Metformin does not reduce luciferase activity of MYC 3′UTR reporter, at the condition of causing MYC protein level changes. Cells were treated with metformin (5 mm, 10 mm) for 24 h, and culture medium was analyzed using the Secrete‐Pair™ Dual Luminescence Assay Kit.

To characterize the post‐transcriptional step at which metformin reduces MYC protein, we treated cells with cycloheximide (CHX) to block protein synthesis or MG132 to block protein degradation (Schneider‐Poetsch *et al*., 2010; Tsubuki *et al*., 1996). We reason that if metformin promotes MYC degradation, blockage of protein degradation by MG132 should abrogate the metformin effect on MYC, while blockage of protein synthesis with CHX should have an inverse effect. We treated cells in standard culture conditions with 10 mm metformin for 24 h, at which time point MYC protein levels were reduced to half (Fig. 3A). As expected, blockage of protein synthesis with CHX resulted in a time‐dependent reduction of MYC protein expression. However, we were unable to rescue MYC expression with MG132 arguing against altered MYC proteasomal degradation by metformin (Fig. 3A,B). Consistently, metformin did not promote the degradation of MYC protein in CHX kinetic experiments (Fig. 3A,B). MYC phosphorylation at threonine 58 is a well‐characterized signal‐promoting MYC degradation (Sears *et al*., 2000; Yeh *et al*., 2004). However, metformin treatment did not alter Thr58 phosphorylation on MYC (p‐MYC) (Fig. 3C). This lack of p‐MYC induction by metformin was also observed in lysates from colony formation assays (Fig. 3D). The above experimental data exclude the possibility of changes in MYC turnover as a consequence of metformin treatment, and suggest that metformin causes reduced MYC protein synthesis. Since microRNA could block MYC protein synthesis by imperfect complementary binding to the 3′‐UTR of the mRNA, we tested this possibility. Under conditions where metformin significantly reduced MYC protein expression, there is no significant change in MYC 3′‐UTR luciferase reporter activity (Fig. 3E). This suggests that the observed changes in MYC protein levels are not mediated by microRNA activity on the 3′‐UTR of MYC mRNA.

**Figure 4:**
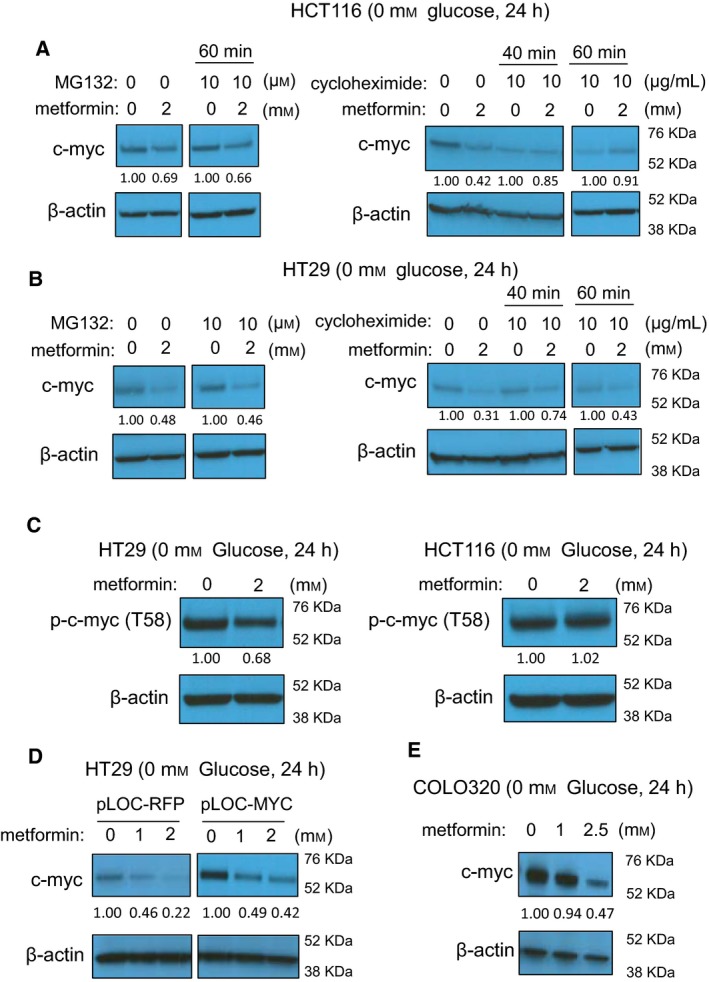
Metformin blocks MYC protein synthesis in glucose‐free media. (A,B) Metformin‐induced MYC reduction is neither rescued by the proteasome inhibitor MG132, nor accelerated by the protein synthesis inhibitor CHX in HCT116 cells (A) and in HT29 cells (B). In A and B, cells were treated with metformin for 24 h (2 mm), in the presence or absence of MG132 (10 μM). (C) Metformin does not enhance the MYC phosphorylation at threonine 58. (D) Metformin reduces the overexpressed MYC protein in HT29 cells established with lentiviral MYC plasmid lacking 5′UTR and 3′UTR. (E) Metformin reduces MYC protein in COLO320 cells that express high level of endogenous MYC due to genomic amplification.

It is possible that in glucose‐free conditions, the regulatory MYC mechanisms stimulated by metformin are different from those active under standard glucose concentrations. As such, we repeated our experiments in glucose‐free conditions. Similar to our data obtained under standard glucose concentrations, we did not observe enhanced degradation of MYC protein in glucose‐free conditions using CHX and MG132 assays (Fig. 4A,B). p‐MYC was not induced by metformin in glucose‐free media, similar to our data under standard glucose concentrations (Fig. 4C). Interestingly, metformin reduced MYC protein expression even when MYC was expressed from a pLOC lentiviral vector, which contains only the MYC coding sequence without UTRs (Fig. 4D). This finding further supports the possibility that MYC regulation by metformin is independent of microRNA activity on the 3′UTR of MYC. Metformin is equally effective in reducing MYC levels in COLO320 cells where MYC is amplified and expressed at high levels (Alitalo *et al*., 1983; Trainer *et al*., 1988) (Fig. 4E). Together, these data strongly suggest that metformin blocks MYC protein synthesis.

### 3.4 Metformin induces widespread inhibition of protein synthesis

**Figure 5:**
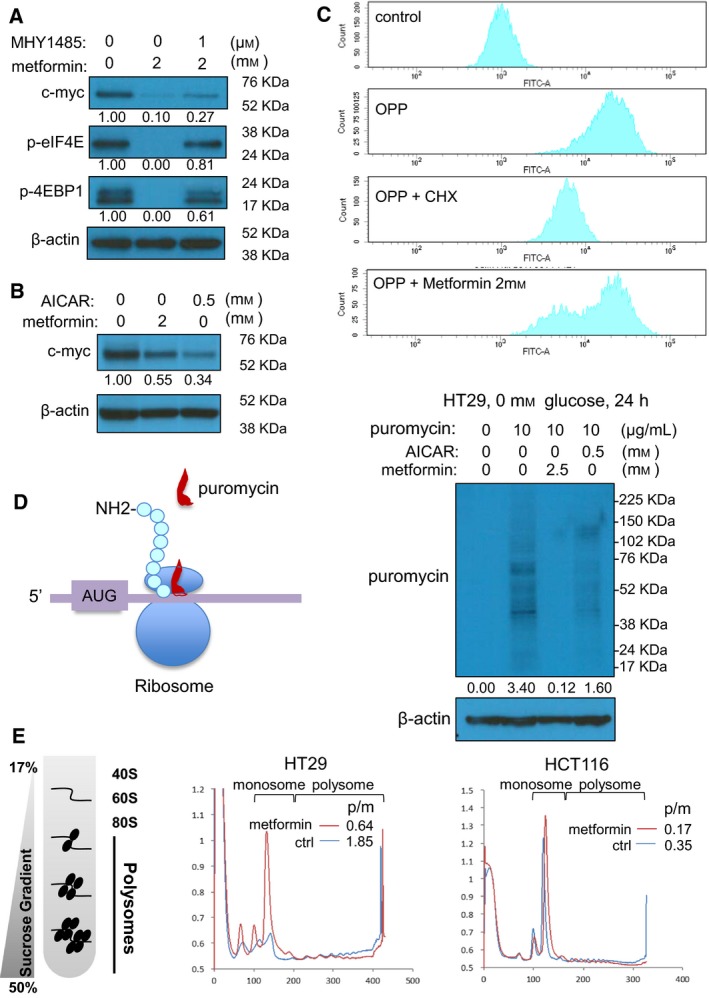
Metformin induces widespread inhibition of protein synthesis. (A) Metformin abrogates the expression of p‐4EBP1 and p‐eIF4E at 24 h, and the effect is partially rescued by coincubation of cells with the mTOR activator MHY1485, in HCT116 cells. (B) The AMPK activator AICAR reduces MYC expression, similar as metformin in HCT116 cells. (C) Metformin blocks protein synthesis, as shown by reduced FITC signal, in HT29 cells stained with OPP, a puromycin analog that incorporated only to newly synthesized proteins. (D) Left panel shows the rational of using ribopuromycylation (puromycin labeling) to monitor protein synthesis, after which the newly synthesized protein can be detected by antibody against puromycin. Metformin strongly blocks protein synthesis, shown by reduced puromycin labeling. AICAR induces a similar yet weaker effect. HT29 cells were treated with metformin (2.5 mm) or AICAR (0.5 mm) for 24 h, and then, the cells were incubated with puromycin (10 μg/mL) for 20 min. Total proteins were isolated and detected by western blot using puromycin antibody. (E) Metformin causes an increase of mRNA association with polysomes and a reduction of mRNA association with monosomes.

AMP‐activated protein kinase (AMPK) and mammalian target of rapamycin (mTOR) signaling have previously been demonstrated to be essential metformin targets that control protein synthesis (Chan *et al*., 2004; Howell *et al*., 2017; Larsson *et al*., 2012). As reported, metformin activated AMPK as reflected by increased p‐ACC (Fig. S5) (Galdieri *et al*., 2016) and inhibited mTOR as shown by decreased p‐4EBP1 and p‐eIF4E (Fig. 5A) (Dowling *et al*., 2007). Similar to metformin, treatment of CRC cells with AICAR, a chemical AMPK activator, leads to a dramatic reduction of MYC protein (Fig. 5B). In contrast, MHY1485, a chemical mTOR activator, partially reversed metformin's effect on p‐4EBP1, p‐eIF4E, and MYC expression (Fig. 5A). These data suggest that metformin reduces MYC expression in CRC cells through AMPK activation and mTOR inhibition.

Because of the general effect of mTOR‐4EBP‐eIF4E signaling in controlling protein synthesis, we tested whether the repression of MYC by metformin represents a global blockage of protein synthesis. To this end, we analyze the newly synthesized proteins by employing a cell‐permeable, puromycin analog O‐propargyl‐puromycin (OPP). The incorporation of OPP into the C terminus of translating polypeptide chains can be subsequently detected via copper‐catalyzed click chemistry using 5 FAM‐Azide (Liu *et al*., 2012). Flow cytometric analysis can then be used to detect in each cell the total ongoing protein synthesis based on 5‐FAM fluorescence, if it is occurring. This method indicated clear fluorescence in control OPP‐treated samples, and as expected, low 5‐FAM signal when translation elongation was blocked with CHX (Fig. 5C). Metformin treatment resulted in a wide histogram peak with hallmarks of both the OPP control and OPP CHX‐treated samples, suggesting that a subpopulation of cells is subject to protein synthesis inhibition (Figs 5C and S6A). We confirmed the metformin effects on protein synthesis using the ribopuromycylation assay, which detects all actively translating ribosomes in the whole‐cell population (David *et al*., 2012; Schmidt *et al*., 2009). After metformin treatment, we incubated HT29 cells with puromycin for 20 min to label newly synthesized proteins (Fig. S6B). Labeled proteins can then be detected by western blot using antibodies against puromycin. In control cells, we observed puromycin‐labeled proteins that correspond to a wide range of sizes and with high intensity (Fig. 5D). In contrast, metformin greatly reduced the levels of nascent puromycin‐labeled proteins (Fig. 5D). The AMPK activator AICAR, which induced a weaker yet more uniform inhibition on protein synthesis (Fig. S7), reduced ribopuromycylation in the whole‐cell population to a lesser extent than metformin (Figs 5D and S8). Co‐treatment with MHY1485 and metformin did not significantly diminish the inhibitory effect of metformin on protein synthesis in either of these assays (Figs S7 and S8). This suggests that partial rescue of mTOR signaling (Fig. 5A), which reverses protein expression of sensitive genes such as MYC, is not strong enough to rescue global protein synthesis.

To further analyze the metformin effect on protein synthesis, we performed polysome profiling to examine the association of mRNA with ribosomes (Chasse *et al*., 2017). Polysome profiling showed a decrease in polyribosomal mRNA, and an increase in abundance of monoribosomal mRNA after metformin treatment. This is a hallmark shift in polysome complexes indicative of translational repression. In HT29 and HCT116 cells, the polysome/monosome ratio reduced from 1.85 (control) to 0.64 (metformin) and from 0.35 (control) to 0.17 (metformin), respectively (Fig. 5E). Taken together, these results suggest that metformin blocks protein synthesis, probably as a consequence of its effect on AMPK and mTOR signaling. However, it is important to note that not all translation disappears in any of these assays upon metformin treatment, which suggests this effect is specific for a subset of mRNAs.

### 3.5 Metformin represses protein synthesis with a preference on cell cycle‐related proteins

**Figure 6:**
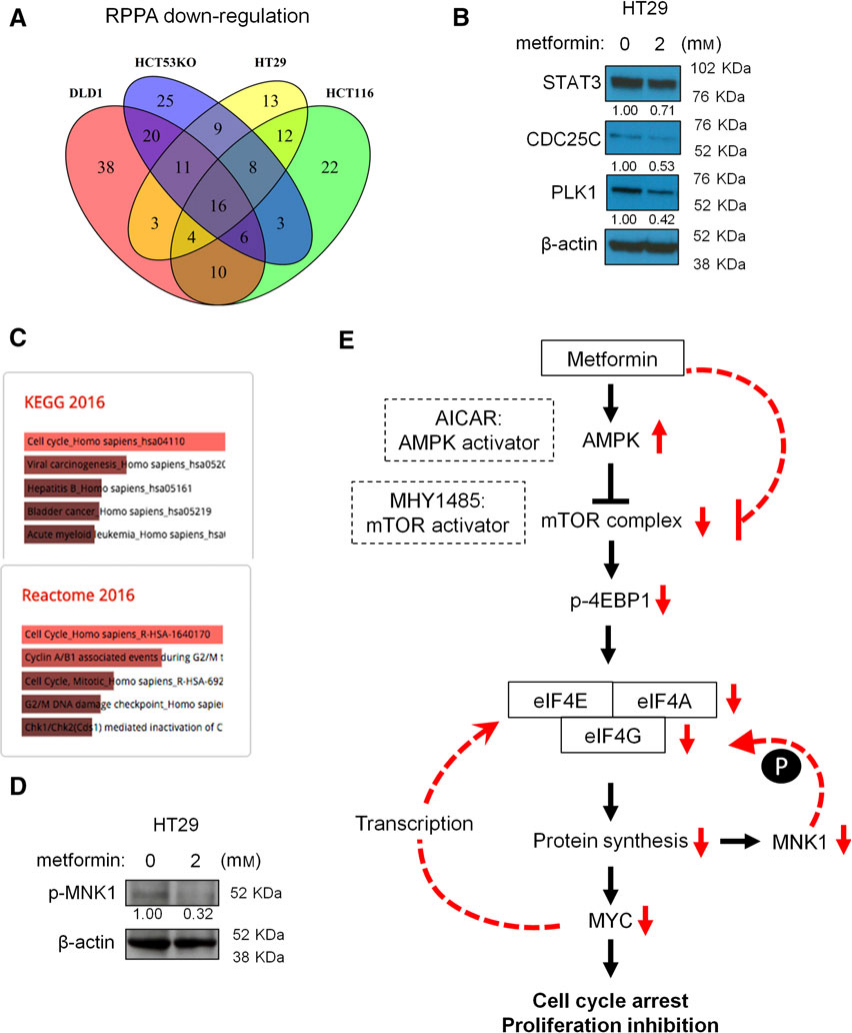
Metformin preferably represses the protein synthesis of cell cycle‐related genes. (A) Venn diagram of proteins that are downregulated by metformin (2 mm for 24 h) in four CRC cell lines identified with reverse‐phase protein array (RPPA). (B) Validation of the overlapping downregulated proteins by western blot. (C) Enrich analysis of the 16 downregulated proteins by RPPA revealed an enrichment of proteins that regulate cell cycle. (D) Metformin reduces the expression of p‐MNK1, concordant with the downregulated MNK1 by metformin in RPPA analysis. (E) A schematic diagram presenting the proposed mechanism of metformin effects.

To test whether metformin inhibits expression of a subset of mRNAs, we sought to identify the panel of proteins affected by metformin using RPPA in four CRC cell lines with biological duplicates of each. Using the criteria that proteins should change in the same direction in both duplicates, we identified 3 upregulated proteins and 16 downregulated proteins in all four cell lines during metformin treatment, including the already demonstrated MYC protein (Fig. 6A and Table S3). We selected several known important proteins in cancer biology, including STAT3, CDC25C, and PLK1, and validated RPPA data by western blotting (Fig. 6B). The RNA level of these genes is not affected by metformin (Fig. S9), suggesting that protein synthesis is responsible for the reduced protein expression. These data are consistent with changes in translation of subsets of mRNAs (Fig. 5C,D,E). Enrichment pathway analysis of the 16 downregulated proteins identified in RPPA revealed proteins that regulate cell cycle (Fig. 6C), indicating preferential inhibition of cell cycle‐related genes by metformin. In addition, MAP kinase‐interacting protein kinase 1 (MNK1), which regulates eIF4E phosphorylation and is bound by eIF4G, is one of the downregulated proteins identified by RPPA. Western blot data showed that p‐MNK1, the activate form of MNK1, was reduced during metformin treatment (Fig. 6D). More recently, MNK1 was reported to regulate mTORC1 signaling by promoting association of mTORC1 with its substrates (Brown and Gromeier, 2017). Thus, reduction of MNK1 by metformin further inhibits protein synthesis by mTORC‐independent (eIF4G‐eIF4E) as well as mTORC‐dependent (mTORC1: substrate) mechanisms (Fig. 6E).

## 4 Discussion

Accumulating evidence supports the beneficial use of metformin in reducing CRC risk and malignancy (Bodmer *et al*., 2010; Currie *et al*., 2009; Evans *et al*., 2005; Tsai *et al*., 2014), but the underlying mechanisms remain unclear. Increased transcription of Wnt target genes by the β‐catenin/TCF complex is an essential mechanism driving CRC carcinogenesis, progression, and metastasis (Cancer Genome Atlas, N, 2012; Fodde and Brabletz, 2007; Liu *et al*., 2000; Phelps *et al*., 2009; Vermeulen *et al*., 2010). It is thus reasonable to suspect that metformin might regulate Wnt signaling to exert beneficial activities in CRC. Indeed, metformin was shown to reduce the size of intestinal polyps in Apc^Min/+^ carcinogenesis mouse model (Tomimoto *et al*., 2008). However, our data revealed an increase, not a decrease, in Wnt activity following metformin treatment. In contrast, MYC protein expression was dramatically reduced by metformin, despite a lack of change at mRNA level (He *et al*., 1998). In the study with Apc^Min/+^ mouse model, metformin did not alter MYC RNA expression, and its effect on MYC protein was not tested (Tomimoto *et al*., 2008). Our data suggest that the beneficial activity of metformin is not by inhibition of MYC transcription, but by inhibition of its translation. Indeed, MYC is one of the most well‐known oncogenes, and comprehensive analysis of gene expression in TCGA clearly revealed a pivotal role for MYC in CRC (Cancer Genome Atlas, N, 2012; Gabay *et al*., 2014).

MYC is a desired but so far ‘undruggable’ target for cancer treatment. Recent insights into its regulation have led to therapeutic opportunities, as exemplified by reducing MYC transcription using BRD4 inhibitors (Filippakopoulos *et al*., 2010; Mertz *et al*., 2011). Compared with these emerging drug developments, metformin has several advantages as a MYC inhibitor: (a) As a first‐line drug treating diabetes, metformin is safe, and its side effects are well characterized. (b) Metformin robustly reduces the protein synthesis of MYC, irrespective of its transcriptional status in many CRC cell lines. In contrast, the BRD4 inhibitor JQ1 only reduces MYC in certain CRC cell types. (c) The short half‐life of MYC protein sensitizes it to protein synthesis inhibition. (d) The oral route of metformin is ideal for diseases of the digestive system. (e) Metformin's effect on MYC is independent of glucose, and not a mere consequence of compensated glycolysis of cells in response to energy loss. These features make metformin an attractive candidate for potential use against many MYC‐driven cancers.

Several previous studies reported MYC regulation by metformin, via either microRNA regulation in breast cancer or protein degradation in prostate cancer (Akinyeke *et al*., 2013; Blandino *et al*., 2012). We acknowledge that metformin is a potent drug with multiple mechanisms, which can be context dependent. However, under the conditions used in our experiments, metformin did not affect MYC 3‐UTR luciferase reporter activity. This was further substantiated by the fact that metformin reduced ectopic expression of MYC from a pLOC expression vector lacking MYC UTR region. Furthermore, we did not observe evidence of increased MYC protein degradation using multiple strategies. These discrepancies with previous studies may be due to different drug concentrations, time points, and cell types. Nevertheless, considering the well‐established effect of metformin in mTOR signaling and protein synthesis, it is reasonable that protein synthesis of MYC would be affected.

We found that metformin causes a broad downregulation of protein synthesis only in a subpopulation of cells, whereas CHX reduced protein synthesis in the whole‐cell population. We suspect that a subpopulation of cells at a specific cell cycle stage is preferentially targeted by metformin. Indeed, RPPA data revealed an enrichment of cell cycle‐related genes including CDC25C and PLK1 in proteins downregulated by metformin, echoing a previous report showing selective translational inhibition of cell cycle regulators such as cyclin E2 and ODC1 by metformin in breast cancer cells (Larsson *et al*., 2012). However, the gene sets regulated by metformin in the previous report are different from the ones that we identified, and whether these genes represent a context‐dependent ‘eIF4E‐sensitive’ signature (Jia *et al*., 2012; Yanagiya *et al*., 2012) remains unresolved.

We propose a mechanism of mTOR‐4EBP‐eIF4E axis in regulating translation of MYC and other genes. Interestingly, 4EBP1 has been reported to selectively block translation of genes encoding cell cycle regulators (Larsson *et al*., 2012). In addition, MNK1, which regulates eIF4E phosphorylation via binding to eIF4G, is one of the downregulated proteins identified by RPPA in our study (Pyronnet *et al*., 1999; Ueda *et al*., 2004). It is possible that MNK1 further regulates eIF4E activity via a positive feedback loop in controlling translational initiation. We were surprised that this potent regulation of cap‐dependent translation could have such a strong effect on subsets of mRNA, but this selectivity has been observed before (Yi *et al*., 2013). Cell cycle genes including MYC usually possess complex 5′‐UTR structure and are more dependent on the unwinding activity of the DEAD‐box RNA helicase eIF4A (Koromilas *et al*., 1992; Morita *et al*., 2015). As a key component of the eIF4F heterotrimeric complex, eIF4E not only performs cap‐binding function, but also stimulates eIF4A helicase activity to initiate translation (Feoktistova *et al*., 2013). It is thus possible that the increased availability of eIF4E selectively stimulates the translation of genes that depend on the unwinding activity of eIF4A, such as cell cycle regulators, but not that of housekeeping genes (Feoktistova *et al*., 2013). MYC is a strong transcriptional activator of eIF4E (Jones *et al*., 1996), and thus, by targeting this eIF4E‐MYC feed‐forward loop (Fig. 6E), metformin could achieve cell cycle arrest and growth inhibition of CRC cells (Lin *et al*., 2009). Additionally, other eIF4E‐independent mechanisms have recently been shown to sustain translation for some mRNAs (Guan *et al*., 2017; Lee *et al*., 2015; Uniacke *et al*., 2012). Indeed, eIF4A inhibition has been reported as a promising strategy to reduce MYC expression in colorectal tumors (Wiegering *et al*., 2015), and the possibility of metformin directly inhibiting eIF4A activity cannot be excluded.

Our study has some limitations. First, although MYC is involved in regulating cancer metabolism, the detailed metabolic changes caused by metformin have not been determined in the current study. Second, we acknowledge that inhibition of protein synthesis by metformin affects multiple proteins, with MYC being just one of them. Third, our findings that metformin regulates the protein synthesis of MYC have not been validated in mouse models. The previous work using Apc^Min/+^ mice revealed AMPK activation and mTOR inhibition, and lack of change on MYC mRNA expression in the polyps of metformin‐treated animals (Tomimoto *et al*., 2008). Whether the expression of MYC protein is reduced in such model remains to be determined.

## 5 Conclusions

Our study demonstrated a mechanism of metformin action in CRC, by blocking translation of the *MYC* oncogene. This mechanism not only explains the beneficial effect of metformin in CRC prevention, but also provides additional molecular insight into why high MYC‐expressing cancer cells, which are usually ‘addicted’ to MYC, are more sensitive to metformin treatment (Javeshghani *et al*., 2012). The use of metformin might thus represent a combinatorial strategy to reverse the resistance of CRC cells with high MYC levels to chemotherapeutic drugs.

## Author contributions

PS performed experiments, analyzed the data, performed statistical tests, and wrote the initial draft. LCR performed the polysome profiling assay. MC performed statistical analyses. MP performed experiments. EK performed experiments and revised the manuscript. HL performed experiments, provided supervision and assistance for writing the manuscript and designing figures and tables, and revised the manuscript. GAC secured funding and provided supervision and assistance for data analysis and interpretation and for writing the manuscript. All authors read and approved the final manuscript.

## Supporting information


**Fig. S1.** Metformin inhibits the growth of HCT116 p53^−/−^ and DLD1 cells.
**Fig. S2.** Metformin arrests cells at G1 phase without increasing subG1 population of HCT116 and HT29 cells.
**Fig. S3.** Metformin inhibits colony formation of HCT116 p53^−/−^ and DLD1 cells in a dose‐dependent manner.
**Fig. S4.** Metformin reduces both the RNA and protein levels of MYC in RKO cells.
**Fig. S5.** Metformin activates AMPK as reflected by increased p‐ACC expression.
**Fig. S6.** (A) Protein synthesis kit flow chart. (B) Puromycin pull‐down assay flow chart.
**Fig. S7.** Metformin blocks protein synthesis in HT29 cells by OPP‐based protein synthesis assay.
**Fig. S8.** Metformin blocks protein synthesis in HT29 cells by ribopuromycylation assay.
**Fig. S9.** Metformin does not reduce the RNA expression level of RPPA candidate genes as shown by qRT‐PCR, at the condition that reduces their protein expression in HCT116 and HT29 cells.
**Table S1.** List of reagents.
**Table S2.** List of primers.
**Table S3.** RPPA analysis identified 16 proteins that are downregulated by metformin in all four cell lines, including the already demonstrated MYC protein.

## References

[mol212384-bib-0001] Akinyeke T , Matsumura S , Wang X , Wu Y , Schalfer ED , Saxena A , Yan W , Logan SK and Li X (2013) Metformin targets c‐MYC oncogene to prevent prostate cancer. Carcinogenesis 34, 2823–2832. 24130167 10.1093/carcin/bgt307 PMC3845895

[mol212384-bib-0002] Alitalo K , Schwab M , Lin CC , Varmus HE and Bishop JM (1983) Homogeneously staining chromosomal regions contain amplified copies of an abundantly expressed cellular oncogene (c‐myc) in malignant neuroendocrine cells from a human colon carcinoma. Proc Natl Acad Sci USA 80, 1707–1711. 6300869 10.1073/pnas.80.6.1707 PMC393672

[mol212384-bib-0003] Blandino G , Valerio M , Cioce M , Mori F , Casadei L , Pulito C , Sacconi A , Biagioni F , Cortese G , Galanti S *et al* (2012) Metformin elicits anticancer effects through the sequential modulation of DICER and c‐MYC. Nat Commun 3, 865. 22643892 10.1038/ncomms1859

[mol212384-bib-0004] Bodmer M , Meier C , Krahenbuhl S , Jick SS and Meier CR (2010) Long‐term metformin use is associated with decreased risk of breast cancer. Diabetes Care 33, 1304–1308. 20299480 10.2337/dc09-1791 PMC2875444

[mol212384-bib-0005] Brown MC and Gromeier M (2017) MNK controls mTORC1: substrate association through regulation of TELO2 binding with mTORC1. Cell Rep 18, 1444–1457. 28178522 10.1016/j.celrep.2017.01.023 PMC5321627

[mol212384-bib-0006] Bunz F , Hwang PM , Torrance C , Waldman T , Zhang Y , Dillehay L , Williams J , Lengauer C , Kinzler KW and Vogelstein B (1999) Disruption of p53 in human cancer cells alters the responses to therapeutic agents. J Clin Invest 104, 263–269. 10430607 10.1172/JCI6863 PMC408422

[mol212384-bib-0007] Cancer Genome Atlas, N . (2012) Comprehensive molecular characterization of human colon and rectal cancer. Nature 487, 330–337. 22810696 10.1038/nature11252 PMC3401966

[mol212384-bib-0008] Chan AY , Soltys CL , Young ME , Proud CG and Dyck JR (2004) Activation of AMP‐activated protein kinase inhibits protein synthesis associated with hypertrophy in the cardiac myocyte. J Biol Chem 279, 32771–32779. 15159410 10.1074/jbc.M403528200

[mol212384-bib-0009] Chasse H , Boulben S , Costache V , Cormier P and Morales J (2017) Analysis of translation using polysome profiling. Nucleic Acids Res 45, e15. 28180329 10.1093/nar/gkw907 PMC5388431

[mol212384-bib-0010] Crowley LC , Christensen ME , Waterhouse NJ (2016) Measuring Survival of Adherent Cells with the Colony‐Forming Assay. Cold Spring Harb Protoc 2016, pdb prot087171. 10.1101/pdb.prot087171 27480717

[mol212384-bib-0011] Currie CJ , Poole CD and Gale EA (2009) The influence of glucose‐lowering therapies on cancer risk in type 2 diabetes. Diabetologia 52, 1766–1777. 19572116 10.1007/s00125-009-1440-6

[mol212384-bib-0012] David A , Dolan BP , Hickman HD , Knowlton JJ , Clavarino G , Pierre P , Bennink JR and Yewdell JW (2012) Nuclear translation visualized by ribosome‐bound nascent chain puromycylation. J Cell Biol 197, 45–57. 22472439 10.1083/jcb.201112145 PMC3317795

[mol212384-bib-0013] Dowling RJ , Zakikhani M , Fantus IG , Pollak M and Sonenberg N (2007) Metformin inhibits mammalian target of rapamycin‐dependent translation initiation in breast cancer cells. Cancer Res 67, 10804–10812. 18006825 10.1158/0008-5472.CAN-07-2310

[mol212384-bib-0014] Evans JM , Donnelly LA , Emslie‐Smith AM , Alessi DR and Morris AD (2005) Metformin and reduced risk of cancer in diabetic patients. BMJ 330, 1304–1305. 15849206 10.1136/bmj.38415.708634.F7 PMC558205

[mol212384-bib-0015] Feoktistova K , Tuvshintogs E , Do A and Fraser CS (2013) Human eIF4E promotes mRNA restructuring by stimulating eIF4A helicase activity. Proc Natl Acad Sci USA 110, 13339–13344. 23901100 10.1073/pnas.1303781110 PMC3746923

[mol212384-bib-0016] Filippakopoulos P , Qi J , Picaud S , Shen Y , Smith WB , Fedorov O , Morse EM , Keates T , Hickman TT , Felletar I *et al* (2010) Selective inhibition of BET bromodomains. Nature 468, 1067–1073. 20871596 10.1038/nature09504 PMC3010259

[mol212384-bib-0017] Fodde R and Brabletz T (2007) Wnt/beta‐catenin signaling in cancer stemness and malignant behavior. Curr Opin Cell Biol 19, 150–158. 17306971 10.1016/j.ceb.2007.02.007

[mol212384-bib-0018] Gabay M , Li Y , Felsher DW (2014) MYC activation is a hallmark of cancer initiation and maintenance. Cold Spring Harb Perspect Med 4, a014241. 24890832 10.1101/cshperspect.a014241 PMC4031954

[mol212384-bib-0019] Galdieri L , Gatla H , Vancurova I and Vancura A (2016) Activation of AMP‐activated protein kinase by metformin induces protein acetylation in prostate and ovarian cancer cells. J Biol Chem 291, 25154–25166. 27733682 10.1074/jbc.M116.742247 PMC5122782

[mol212384-bib-0020] Guan BJ , van Hoef V , Jobava R , Elroy‐Stein O , Valasek LS , Cargnello M , Gao XH , Krokowski D , Merrick WC , Kimball SR *et al* (2017) A Unique ISR program determines cellular responses to chronic stress. Mol Cell 68, 885–900 e886. 29220654 10.1016/j.molcel.2017.11.007 PMC5730339

[mol212384-bib-0021] He TC , Sparks AB , Rago C , Hermeking H , Zawel L , da Costa LT , Morin PJ , Vogelstein B and Kinzler KW (1998) Identification of c‐MYC as a target of the APC pathway. Science 281, 1509–1512. 9727977 10.1126/science.281.5382.1509

[mol212384-bib-0022] Higurashi T , Hosono K , Takahashi H , Komiya Y , Umezawa S , Sakai E , Uchiyama T , Taniguchi L , Hata Y , Uchiyama S *et al* (2016) Metformin for chemoprevention of metachronous colorectal adenoma or polyps in post‐polypectomy patients without diabetes: a multicentre double‐blind, placebo‐controlled, randomised phase 3 trial. Lancet Oncol 17, 475–483. 26947328 10.1016/S1470-2045(15)00565-3

[mol212384-bib-0023] Howell JJ , Hellberg K , Turner M , Talbott G , Kolar MJ , Ross DS , Hoxhaj G , Saghatelian A , Shaw RJ and Manning BD (2017) Metformin inhibits hepatic mTORC1 signaling via dose‐dependent mechanisms involving AMPK and the TSC complex. Cell Metab 25, 463–471. 28089566 10.1016/j.cmet.2016.12.009 PMC5299044

[mol212384-bib-0024] Javeshghani S , Zakikhani M , Austin S , Bazile M , Blouin MJ , Topisirovic I , St‐Pierre J and Pollak MN (2012) Carbon source and myc expression influence the antiproliferative actions of metformin. Cancer Res 72, 6257–6267. 23041548 10.1158/0008-5472.CAN-12-2907

[mol212384-bib-0025] Jia Y , Polunovsky V , Bitterman PB and Wagner CR (2012) Cap‐dependent translation initiation factor eIF4E: an emerging anticancer drug target. Med Res Rev 32, 786–814. 22495651 10.1002/med.21260 PMC7168506

[mol212384-bib-0026] Jones RM , Branda J , Johnston KA , Polymenis M , Gadd M , Rustgi A , Callanan L and Schmidt EV (1996) An essential E box in the promoter of the gene encoding the mRNA cap‐binding protein (eukaryotic initiation factor 4E) is a target for activation by c‐myc. Mol Cell Biol 16, 4754–4764. 8756633 10.1128/mcb.16.9.4754 PMC231476

[mol212384-bib-0027] Koromilas AE , Lazaris‐Karatzas A and Sonenberg N (1992) mRNAs containing extensive secondary structure in their 5′ non‐coding region translate efficiently in cells overexpressing initiation factor eIF‐4E. EMBO J 11, 4153–4158. 1396596 10.1002/j.1460-2075.1992.tb05508.x PMC556925

[mol212384-bib-0028] Larsson O , Morita M , Topisirovic I , Alain T , Blouin MJ , Pollak M and Sonenberg N (2012) Distinct perturbation of the translatome by the antidiabetic drug metformin. Proc Natl Acad Sci USA 109, 8977–8982. 22611195 10.1073/pnas.1201689109 PMC3384216

[mol212384-bib-0029] Lee AS , Kranzusch PJ and Cate JH (2015) eIF3 targets cell‐proliferation messenger RNAs for translational activation or repression. Nature 522, 111–114. 25849773 10.1038/nature14267 PMC4603833

[mol212384-bib-0030] Lin CJ , Malina A and Pelletier J (2009) c‐Myc and eIF4F constitute a feedforward loop that regulates cell growth: implications for anticancer therapy. Cancer Res 69, 7491–7494. 19773439 10.1158/0008-5472.CAN-09-0813

[mol212384-bib-0031] Liu W , Dong X , Mai M , Seelan RS , Taniguchi K , Krishnadath KK , Halling KC , Cunningham JM , Boardman LA , Qian C *et al* (2000) Mutations in AXIN2 cause colorectal cancer with defective mismatch repair by activating beta‐catenin/TCF signalling. Nat Genet 26, 146–147. 11017067 10.1038/79859

[mol212384-bib-0032] Liu J , Xu Y , Stoleru D and Salic A (2012) Imaging protein synthesis in cells and tissues with an alkyne analog of puromycin. Proc Natl Acad Sci USA 109, 413–418. 22160674 10.1073/pnas.1111561108 PMC3258597

[mol212384-bib-0033] Marini C , Bianchi G , Buschiazzo A , Ravera S , Martella R , Bottoni G , Petretto A , Emionite L , Monteverde E , Capitanio S *et al* (2016) Divergent targets of glycolysis and oxidative phosphorylation result in additive effects of metformin and starvation in colon and breast cancer. Sci Rep 6, 19569. 26794854 10.1038/srep19569 PMC4726140

[mol212384-bib-0034] Mertz JA , Conery AR , Bryant BM , Sandy P , Balasubramanian S , Mele DA , Bergeron L and Sims RJ 3rd (2011) Targeting MYC dependence in cancer by inhibiting BET bromodomains. Proc Natl Acad Sci USA 108, 16669–16674. 21949397 10.1073/pnas.1108190108 PMC3189078

[mol212384-bib-0035] Morita M , Gravel SP , Hulea L , Larsson O , Pollak M , St‐Pierre J and Topisirovic I (2015) mTOR coordinates protein synthesis, mitochondrial activity and proliferation. Cell Cycle 14, 473–480. 25590164 10.4161/15384101.2014.991572 PMC4615141

[mol212384-bib-0036] Ohtsuka M , Ling H , Ivan C , Pichler M , Matsushita D , Goblirsch M , Stiegelbauer V , Shigeyasu K , Zhang X , Chen M *et al* (2016) H19 noncoding RNA, an independent prognostic factor, regulates essential Rb‐E2F and CDK8‐beta‐catenin signaling in colorectal cancer. EBioMedicine 13, 113–124. 27789274 10.1016/j.ebiom.2016.10.026 PMC5264449

[mol212384-bib-0037] Pereboom TC , Bondt A , Pallaki P , Klasson TD , Goos YJ , Essers PB , Groot Koerkamp MJ , Gazda HT , Holstege FC , Costa LD *et al* (2014) Translation of branched‐chain aminotransferase‐1 transcripts is impaired in cells haploinsufficient for ribosomal protein genes. Exp Hematol 42(394–403), e394. 10.1016/j.exphem.2013.12.010 24463277

[mol212384-bib-0038] Phelps RA , Chidester S , Dehghanizadeh S , Phelps J , Sandoval IT , Rai K , Broadbent T , Sarkar S , Burt RW and Jones DA (2009) A two‐step model for colon adenoma initiation and progression caused by APC loss. Cell 137, 623–634. 19450512 10.1016/j.cell.2009.02.037 PMC2706149

[mol212384-bib-0039] Pyronnet S , Imataka H , Gingras AC , Fukunaga R , Hunter T and Sonenberg N (1999) Human eukaryotic translation initiation factor 4G (eIF4G) recruits mnk1 to phosphorylate eIF4E. EMBO J 18, 270–279. 9878069 10.1093/emboj/18.1.270 PMC1171121

[mol212384-bib-0040] Schmidt EK , Clavarino G , Ceppi M and Pierre P (2009) SUnSET, a nonradioactive method to monitor protein synthesis. Nat Methods 6, 275–277. 19305406 10.1038/nmeth.1314

[mol212384-bib-0041] Schneider‐Poetsch T , Ju J , Eyler DE , Dang Y , Bhat S , Merrick WC , Green R , Shen B and Liu JO (2010) Inhibition of eukaryotic translation elongation by cycloheximide and lactimidomycin. Nat Chem Biol 6, 209–217. 20118940 10.1038/nchembio.304 PMC2831214

[mol212384-bib-0042] Sears R , Nuckolls F , Haura E , Taya Y , Tamai K and Nevins JR (2000) Multiple Ras‐dependent phosphorylation pathways regulate Myc protein stability. Genes Dev 14, 2501–2514. 11018017 10.1101/gad.836800 PMC316970

[mol212384-bib-0043] Siegel RL , Miller KD , Fedewa SA , Ahnen DJ , Meester RGS , Barzi A and Jemal A (2017a) Colorectal cancer statistics, 2017. CA Cancer J Clin 67, 177–193. 28248415 10.3322/caac.21395

[mol212384-bib-0044] Siegel RL , Miller KD and Jemal A (2017b) Cancer statistics, 2017. CA Cancer J Clin 67, 7–30. 28055103 10.3322/caac.21387

[mol212384-bib-0045] Thent ZC , Zaidun NH , Azmi MF , Senin MI , Haslan H and Salehuddin R (2017) Is metformin a therapeutic paradigm for colorectal cancer: Insight into the molecular pathway? Curr Drug Targets 18, 734–750. 27919208 10.2174/1389450118666161205125548

[mol212384-bib-0046] Tomimoto A , Endo H , Sugiyama M , Fujisawa T , Hosono K , Takahashi H , Nakajima N , Nagashima Y , Wada K , Nakagama H *et al* (2008) Metformin suppresses intestinal polyp growth in ApcMin/+ mice. Cancer Sci 99, 2136–2141. 18803638 10.1111/j.1349-7006.2008.00933.x PMC11159964

[mol212384-bib-0047] Trainer DL , Kline T , McCabe FL , Faucette LF , Feild J , Chaikin M , Anzano M , Rieman D , Hoffstein S , Li DJ *et al* (1988) Biological characterization and oncogene expression in human colorectal carcinoma cell lines. Int J Cancer 41, 287–296. 3338874 10.1002/ijc.2910410221

[mol212384-bib-0048] Tsai MJ , Yang CJ , Kung YT , Sheu CC , Shen YT , Chang PY , Huang MS and Chiu HC (2014) Metformin decreases lung cancer risk in diabetic patients in a dose‐dependent manner. Lung Cancer 86, 137–143. 25267165 10.1016/j.lungcan.2014.09.012

[mol212384-bib-0049] Tsubuki S , Saito Y , Tomioka M , Ito H and Kawashima S (1996) Differential inhibition of calpain and proteasome activities by peptidyl aldehydes of di‐leucine and tri‐leucine. J Biochem 119, 572–576. 8830056 10.1093/oxfordjournals.jbchem.a021280

[mol212384-bib-0050] Ueda T , Watanabe‐Fukunaga R , Fukuyama H , Nagata S and Fukunaga R (2004) Mnk2 and Mnk1 are essential for constitutive and inducible phosphorylation of eukaryotic initiation factor 4E but not for cell growth or development. Mol Cell Biol 24, 6539–6549. 15254222 10.1128/MCB.24.15.6539-6549.2004 PMC444855

[mol212384-bib-0051] Uniacke J , Holterman CE , Lachance G , Franovic A , Jacob MD , Fabian MR , Payette J , Holcik M , Pause A and Lee S (2012) An oxygen‐regulated switch in the protein synthesis machinery. Nature 486, 126–129. 22678294 10.1038/nature11055 PMC4974072

[mol212384-bib-0052] Vermeulen L , De Sousa EMF , van der Heijden M , Cameron K , de Jong JH , Borovski T , Tuynman JB , Todaro M , Merz C , Rodermond H *et al* (2010) Wnt activity defines colon cancer stem cells and is regulated by the microenvironment. Nat Cell Biol 12, 468–476. 20418870 10.1038/ncb2048

[mol212384-bib-0053] Wiegering A , Uthe FW , Jamieson T , Ruoss Y , Huttenrauch M , Kuspert M , Pfann C , Nixon C , Herold S , Walz S *et al* (2015) Targeting translation initiation bypasses signaling crosstalk mechanisms that maintain high MYC levels in colorectal cancer. Cancer Discov 5, 768–781. 25934076 10.1158/2159-8290.CD-14-1040 PMC5166973

[mol212384-bib-0054] Yanagiya A , Suyama E , Adachi H , Svitkin YV , Aza‐Blanc P , Imataka H , Mikami S , Martineau Y , Ronai ZA and Sonenberg N (2012) Translational homeostasis via the mRNA cap‐binding protein, eIF4E. Mol Cell 46, 847–858. 22578813 10.1016/j.molcel.2012.04.004 PMC4085128

[mol212384-bib-0055] Yeh E , Cunningham M , Arnold H , Chasse D , Monteith T , Ivaldi G , Hahn WC , Stukenberg PT , Shenolikar S , Uchida T *et al* (2004) A signalling pathway controlling c‐Myc degradation that impacts oncogenic transformation of human cells. Nat Cell Biol 6, 308–318. 15048125 10.1038/ncb1110

[mol212384-bib-0056] Yi T , Papadopoulos E , Hagner PR and Wagner G (2013) Hypoxia‐inducible factor‐1alpha (HIF‐1alpha) promotes cap‐dependent translation of selective mRNAs through up‐regulating initiation factor eIF4E1 in breast cancer cells under hypoxia conditions. J Biol Chem 288, 18732–18742. 23667251 10.1074/jbc.M113.471466 PMC3696647

