# Supplementary material for: Metformin blocks MYC protein synthesis in colorectal cancer via mTOR‐4EBP‐eIF4E and MNK1‐eIF4G‐eIF4E signaling
Source: Mol Oncol. 2018 Oct 15;12(11):1856–70. doi: 10.1002/1878-0261.12384 (PMC6210051; doi:10.1002/1878-0261.12384)
Supplement: Supplementary file 1 — Fig. S1. Metformin inhibits the growth of HCT116 p53−/− and DLD1 cells. Fig. S2. Metformin arrests cells at G1 phase without increasing subG1 population of HCT116 and HT29 cells. Fig. S3. Metformin inhibits colony formation of HCT116 p53−/− and DLD1 cells in a dose‐dependent manner. Fig. S4. Metformin reduces both the RNA and protein levels of MYC in RKO cells. Fig. S5. Metformin activates AMPK as reflected by increased p‐ACC expression. Fig. S6. (A) Protein synthesis kit flow chart. (B) Puromycin pull‐down assay flow chart. Fig. S7. Metformin blocks protein synthesis in HT29 cells by OPP‐based protein synthesis assay. Fig. S8. Metformin blocks protein synthesis in HT29 cells by ribopuromycylation assay. Fig. S9. Metformin does not reduce the RNA expression level of RPPA candidate genes as shown by qRT‐PCR, at the condition that reduces their protein expression in HCT116 and HT29 cells. Table S1. List of reagents. Table S2. List of primers. Table S3. RPPA analysis identified 16 proteins that are downregulated by metformin in all four cell lines, including the already demonstrated MYC protein. [file MOL2-12-1856-s001.pdf]

Supporting information for the manuscript "Metformin blocks MYC protein synthesis in colorectal cancer via mTOR-4EBP-eIF4E and MNK-1- eIF4G-eIF4E signaling"

Additional Supporting Information may be found online in the supporting information tab for this article as followings:

Figure S1. Metformin inhibit the growth of HCT116 p53-/- and DLD1 cells.

Figure S2. Metformin arrest cells at G1 phase without increasing subG1 population of HCT116 and HT29 cells.

Figure S3. Metformin inhibits colony formation of HCT116 p53-/- and DLD1 cells in a dose-dependent manner.

Figure S4. Metformin reduces both the RNA and protein levels of MYC in RKO cells.

Figure S5. Metformin activates AMPK as reflected by increased p-ACC expression.

Figure S6. (A) Protein synthesis kit flow chart. (B) Puromycin pull-down assay flow chart.

Figure S7. Metformin blocks protein synthesis in HT29 cells by OPP-based protein synthesis assay.

Figure S8. Metformin blocks protein synthesis in HT29 cells by ribopuromycylation assay.

Figure S9. Metformin does not reduce the RNA expression level of RPPA candidate genes as shown by qRT-PCR, at the condition that reduces their protein expression in HCT116 and HT29 cells.

Table S1. List of reagents.

Table S2. List of primers.

Table S3. RPPA analysis identified 16 proteins that are down-regulated by metformin in all four cell lines, including the already demonstrated MYC protein.

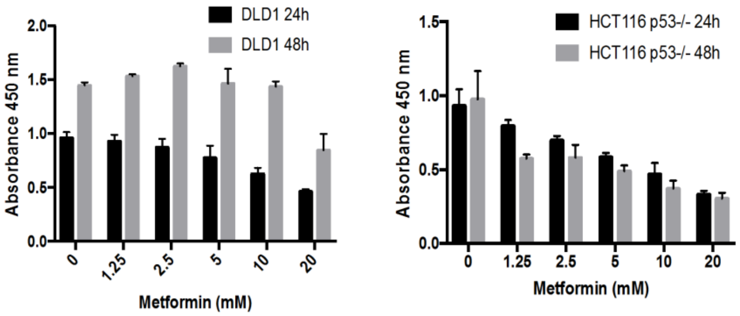

Figure S1. Metformin inhibit the growth of HCT116 p53-/- and DLD1 cells.

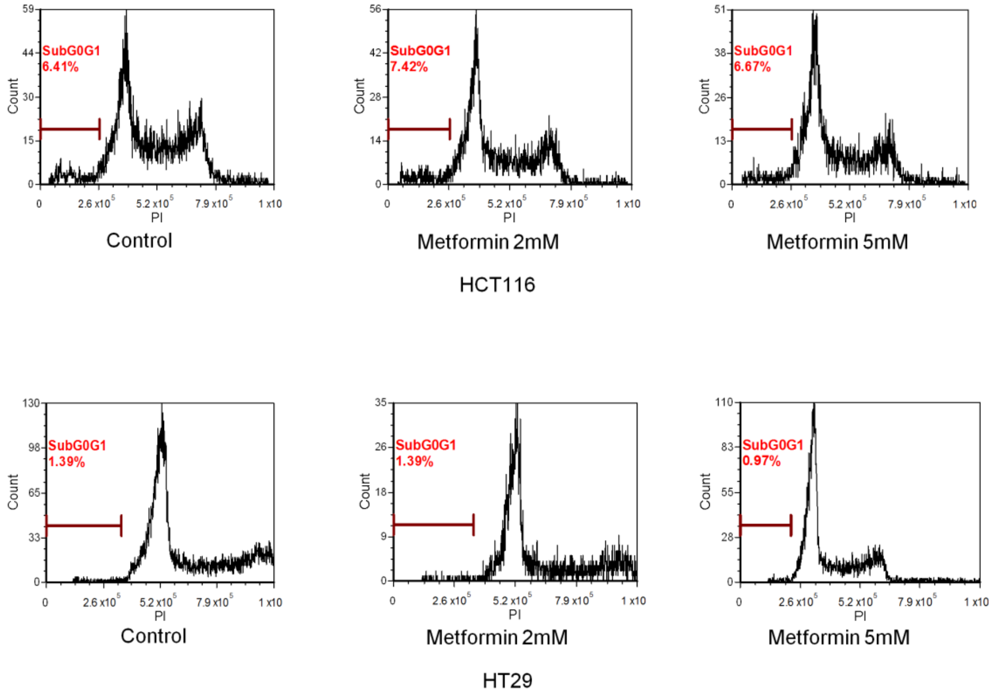

Figure S2. Metformin arrest cells at G1 phase without increasing subG1 population of HCT116 and HT29 cells.

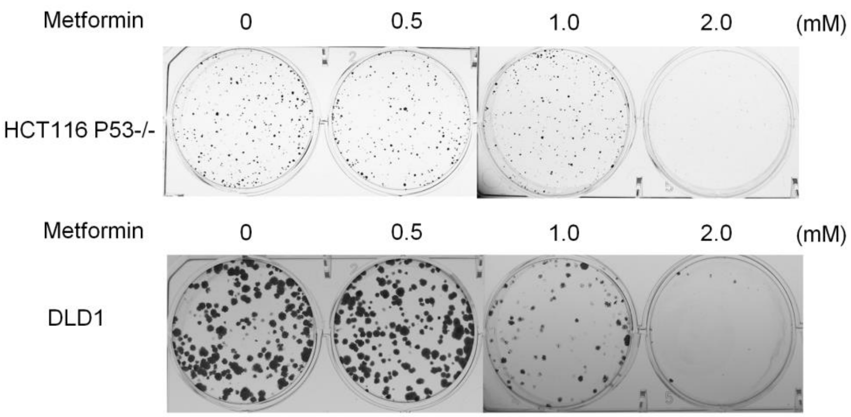

Figure S3. Metformin inhibits colony formation of HCT116 p53-/- and DLD1 cells in a dose-dependent manner.

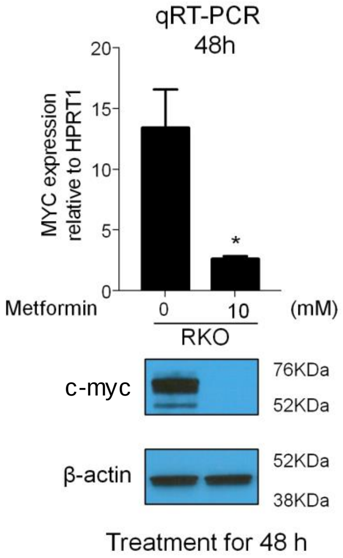

Figure S4. Metformin reduces both the RNA and protein levels of MYC in RKO cells. The qRT-PCR data are presented as the means ± S.D. (n = 4). Student's t-test was used to assess significance. *, $p < 0.05$.

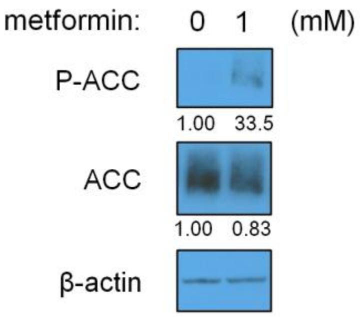

Figure S5. Metformin activates AMPK as reflected by increased p-ACC expression.

A

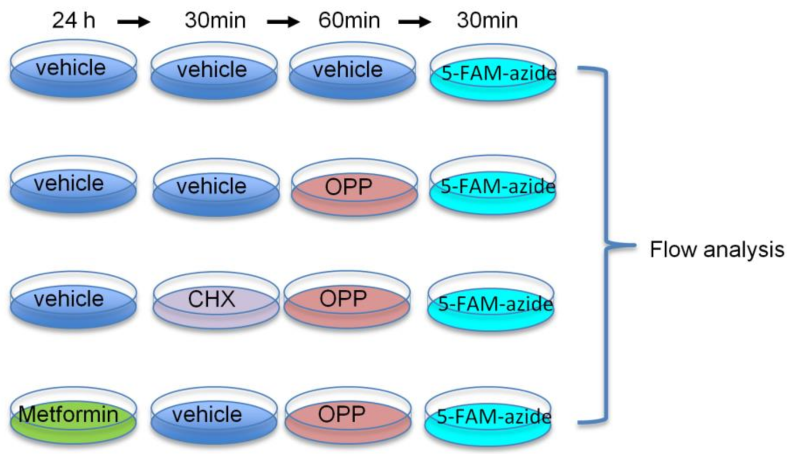

B

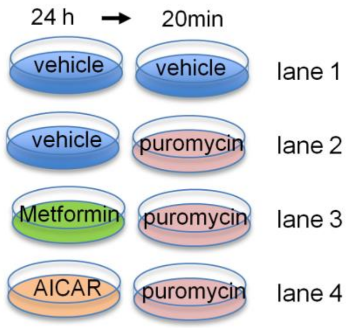

Figure S6. (A) Protein synthesis kit flow chart. (B) Puromycin pull-down assay flow chart.

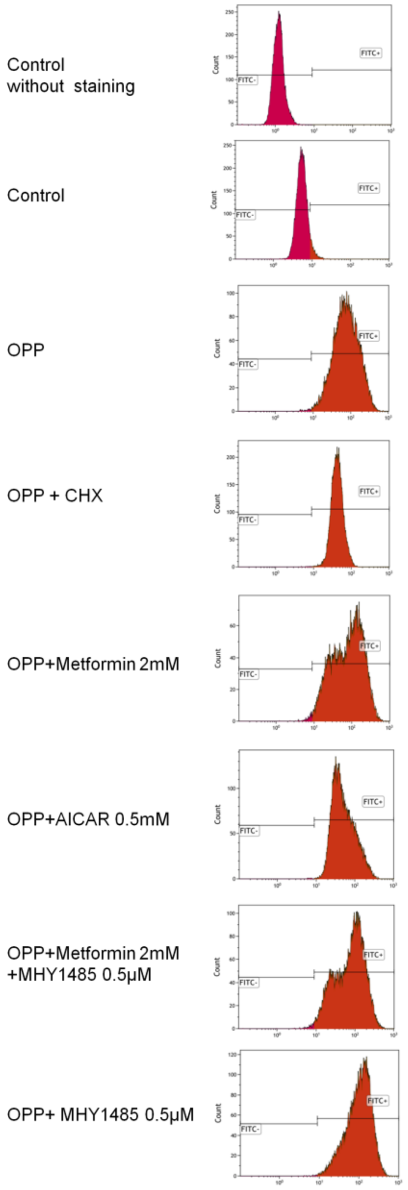

Figure S7. Metformin blocks protein synthesis in HT29 cells by OPP-based protein synthesis assay. HT29 cells were treated with Metformin (2 mM), AICAR (0.5 mM), MHY1485 (1 μM), or Metformin (2mM) + MHY1485 (1 μM), in glucose-free medium for 24 h, and then the cells were collected for OPP assay according to the manufacturer's instructions using Protein Synthesis Assay Kit.

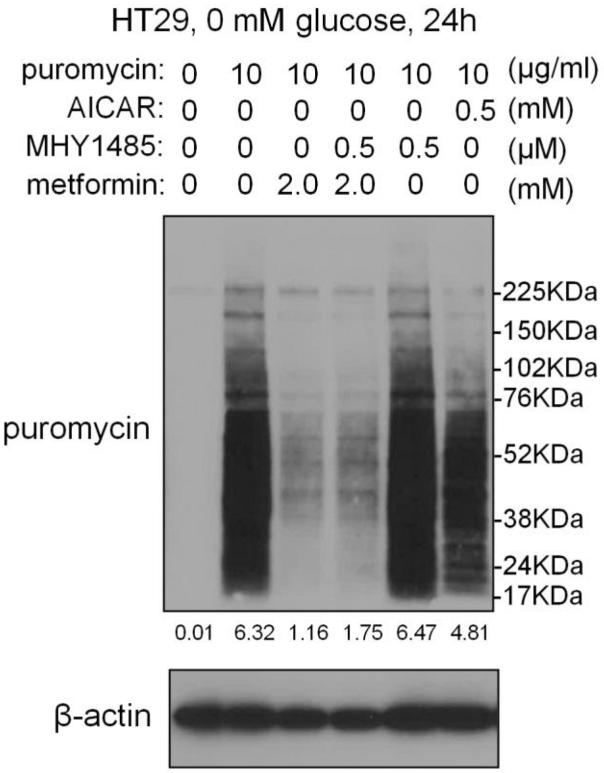

Figure S8. Metformin blocks protein synthesis in HT29 cells by ribopuromycylation assay. AICAR induces a similar yet weaker inhibitory effect. HT29 cells were treated as described in glucose-free medium for 24 h, and then the cells were incubated with puromycin (10 μg/ml) for 20 min. Total proteins were isolated, and detected by western blot using puromycin antibody.

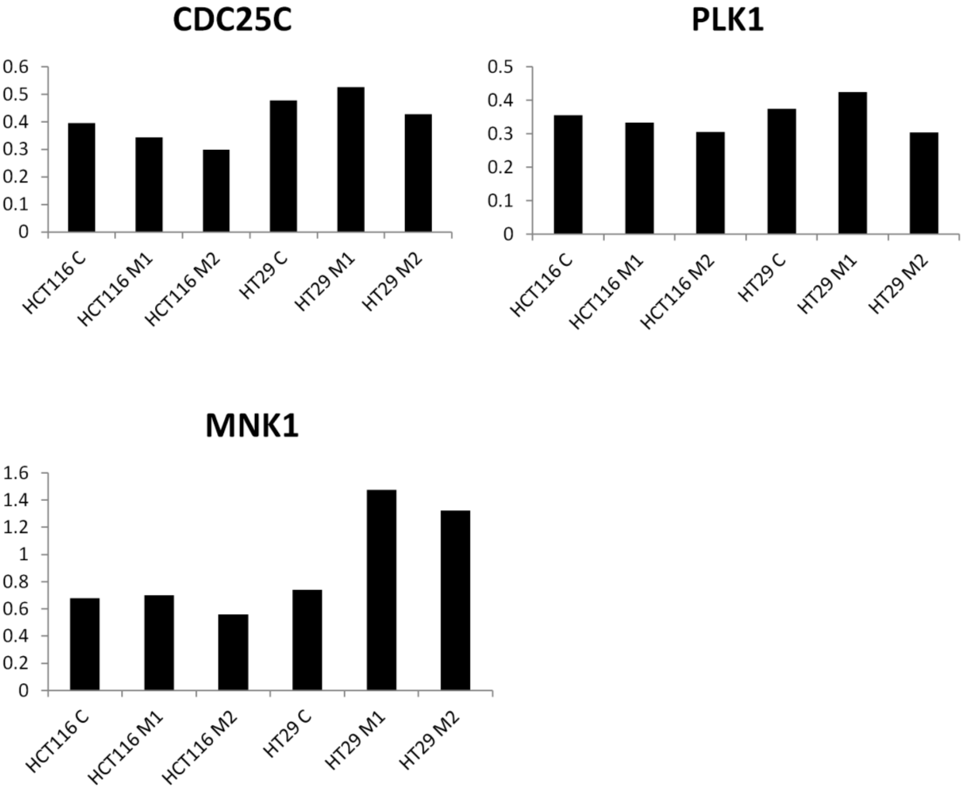

Figure S9. Metformin does not reduce the RNA expression level of RPPA candidate genes as shown by qRT-PCR, at the concentration that reduces their protein expression in HCT116 and HT29 cells.

Table S1. List of reagents used in this study.

**Chemicals**	**Catalog No.**	**Manufacturer**
Metformin hydrochloride	AB120847	Abcam (Cambridge, MA, USA)
Cycloheximide	01810	Sigma-Aldrich (St. Louis, MO, USA)
AICAR	A9978	Sigma-Aldrich (St. Louis, MO, USA)
Dorsomorphin	P5499	Sigma-Aldrich (St. Louis, MO, USA)
Polybrene	H9268	Sigma-Aldrich (St. Louis, MO, USA)
Geneticin	A1720	Sigma-Aldrich (St. Louis, MO, USA)
MG-132	474787	EMD Millipore (Billerica, MA, USA)
MHY1485	500554	EMD Millipore (Billerica, MA, USA)
Rapamycin	553210	EMD Millipore (Billerica, MA, USA)
**Primary Antibodies**	**Catalog No.**	**Manufacturer**
β-actin	A1978	Sigma-Aldrich (St. Louis, MO, USA)
c-Myc	#5741	EMD Millipore (Billerica, MA, USA)
Phospho-c-Myc (T58)	AB185655	Abcam (Cambridge, MA, USA)
AMPKα1	#3195	Cell Signaling Technology (Beverly, MA, USA)
AMPKα2	#9562	Cell Signaling Technology (Beverly, MA, USA)
Phospho-eIF4E(Ser209)	#9741	Cell Signaling Technology (Beverly, MA, USA)
Phospho-4E-BP1 (Thr37/46)	#2855	Cell Signaling Technology (Beverly, MA, USA)
PLK1	#4513	Cell Signaling Technology (Beverly, MA, USA)
Phospho-MNK1	#2111	Cell Signaling Technology (Beverly, MA, USA)
Phospho-CDC25C	#4688	Cell Signaling Technology (Beverly, MA, USA)
Puromycin	EQ0001	Kerafast (Boston, MA,USA)
**Secondary Antibodies**	**Catalog No.**	**Manufacturer**
Anti-Rabbit IgG	A6154	Sigma-Aldrich (St. Louis, MO, USA)
Anti-Mouse IgG	A4416	Sigma-Aldrich (St. Louis, MO, USA)

Table S2. List of primers used in this study.

**PCR primer**	**Primer Sequence**
MYC Forward	CAGCTGCTTAGACGCTGGATT
MYC Reverse	GTAGAAATACGGCTGCACCGA
PLK1 Forward	CACCAGCACGTCGTAGGATTC
PLK1 Reverse	CCGTAGGTAGTATCGGGCCTC
MKNK1 Forward	GCTGACCTCTGAATTGCTTGG
MKNK1 Reverse	TCGATGATTTTGACGGCATACTC
CDC25C Forward	TCTACGGAACTCTTCTCATCCAC
CDC25C Reverse	TCCAGGAGCAGGTTTAACATTTT

Table S3. RPPA analysis identified 16 proteins that are down-regulated by metformin in all four cell lines, including the already demonstrated MYC protein.

Gene name	DLD1	HCT116	HCT116 p53-/-	HT29
PLK1	0.45	0.71	0.51	0.41
EEF2K	0.39	0.75	0.54	0.48
RB1	0.26	0.57	0.41	0.49
FOXM1	0.50	0.86	0.57	0.49
CDC25C	0.75	0.76	0.60	0.52
CHEK1	0.75	0.74	0.79	0.64
CDK1	0.68	0.61	0.74	0.65
MYT1	0.77	0.87	0.83	0.68
MYC	0.73	0.91	0.56	0.68
MKNK1	0.67	0.58	0.58	0.72
SRC	0.72	0.77	0.49	0.72
BRD4	0.64	0.88	0.72	0.73
CHEK2	0.81	0.88	0.79	0.84
XPA	0.84	0.81	0.74	0.85
STAT3	0.51	0.79	0.71	0.87
RPS6KB1	0.57	0.80	0.64	0.91
